# KSGP 3.1: improved taxonomic annotation of Archaea communities using LotuS2, the genome taxonomy database and RNAseq data

**DOI:** 10.1093/ismeco/ycaf094

**Published:** 2025-06-03

**Authors:** Alastair Grant, Abdullah Aleidan, Charli S Davies, Solomon C Udochi, Joachim Fritscher, Mohammad Bahram, Falk Hildebrand

**Affiliations:** School of Environmental Science, University of East Anglia, Norwich NR4 7TJ, United Kingdom; School of Environmental Science, University of East Anglia, Norwich NR4 7TJ, United Kingdom; Zoology Department, College of Sciences, King Saud University, Riyadh 11451, Saudi Arabia; School of Environmental Science, University of East Anglia, Norwich NR4 7TJ, United Kingdom; Department of Biological and Environmental Science, University of Jyväskylä, 40014 Jyväskylän yliopisto, Finland; School of Environmental Science, University of East Anglia, Norwich NR4 7TJ, United Kingdom; The Metals Company, 1111 West Hastings Street, Vancouver, BC V6E 2J3, Canada; Quadram Institute, Norwich Research Park, Norwich NR4 7UQ, United Kingdom; Earlham Institute, Norwich Research Park, Norwich NR4 7UZ, United Kingdom; Department of Ecology, Swedish University of Agricultural Sciences, Box 7044, 75007 Uppsala, Sweden; Department of Botany, Institute of Ecology and Earth Sciences, University of Tartu, 50409 Tartu linn, Tartumaa, Estonia; Department of Agroecology, Aarhus University, Forsøgsvej 1, 4200 Slagelse, Denmark; Quadram Institute, Norwich Research Park, Norwich NR4 7UQ, United Kingdom; Earlham Institute, Norwich Research Park, Norwich NR4 7UZ, United Kingdom

**Keywords:** 16S rRNA, GTDB, SILVA, taxonomic databases, metabarcoding, metatranscriptomics, taxonomic annotation, RNAseq, marine sediment, estuary

## Abstract

Taxonomic annotation is a substantial challenge for Archaea metabarcoding. A limited number of reference sequences are available; a substantial fraction of phylogenetic diversity is not fully characterized; widely used databases do not reflect current archaeal taxonomy and contain mislabelled sequences. We address these gaps with a systematic and tractable approach based around the Genome Taxonomy Database (GTDB) combined with the eukaryote PR2 and MIDORI mitochondrial databases. After removing incongruent, chimeric and duplicate SSU sequences, this combination (*GTDB+*) provides a small improvement in annotation of a set of estuarine Archaea Operational Taxonomic Units (OTUs) compared to SILVA. We add to this a collection of near full length rRNA sequences and the prokaryote SSU sequences in SILVA, creating a new reference database, KSGP (***K***arst, ***S***ilva, ***G***TDB, and **P**R2). The additional sequences are (re-)annotated using three different approaches. The most conservative, using lowest common ancestor, gives a further small improvement. Annotation using SINTAX increases Class and Order assignments by 2.7 and 4.2 times over SILVA, although this may include some “lumping” of un-named and named clades. Still further improvement can be made using similarity based clustering to group database sequences into putative taxa at all taxonomic levels, assigning 60% and 41% of Archaea OTUs to putative family and genus level taxa respectively. GTDB without cleaning and GreenGenes2 both perform poorly and cannot be recommended for use with Archaea. We make the GTDB+ and KSGP databases available at ksgp.earlham.ac.uk; integrate them into a metabarcoding pipeline, LotuS2 and outline their use to annotate Archaea OTUs and metatranscriptomic data.

## Graphical Abstract

**Graphical Abstract:**
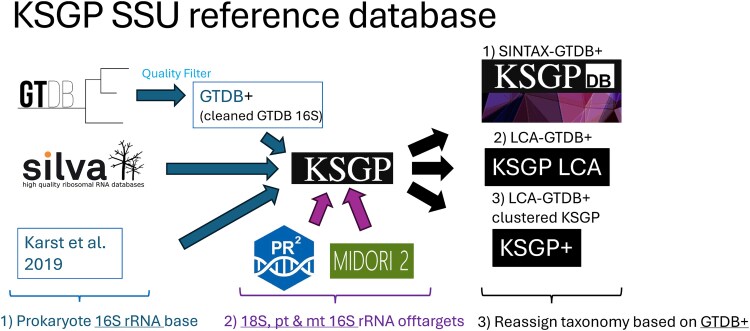

## Introduction

Archaea were initially viewed as extremophiles, but are now recognized as ubiquitous, diverse and sometimes common in benign environments, carrying out important biogeochemical processes including methanogenesis and nitrification [1–5]. However metabarcoding methods for Archaea, particularly taxonomic annotation, are not as well established as for bacteria [6, 7]. Important reference databases such as SILVA [8], Greengenes [9, 10], and RDP [11] contain archaeal sequences in much lower numbers than bacteria. These are often incompletely annotated and archaeal taxonomy lags behind the much more well-defined bacterial taxonomy [12, 13]. NCBI RefSeq reference small subunit (SSU) sequences suffer from taxonomic mis-annotations [14] and Edgar [15] estimated that ⁓17% of taxonomic annotations in SILVA and Greengenes are incorrect. It is unlikely that annotations for the much less well studied Archaea will be more reliable than this average. In addition, there is substantial discordance between archaeal phylogeny and NCBI taxonomy [13] and “universal” 16S rRNA gene primers are often biassed against Archaea, especially newly discovered, lineages [7, 16, 17].

A route to improve this is provided by the standardized archaeal phylogeny and taxonomy constructed from conserved single copy marker genes in the prokaryote Genome Taxonomy Data Base [hereafter GTDB; [13, 18]]. This assigns all available Archaea genomes, including metagenomic assembled genomes (MAGs), to “species clusters” and normalizes taxonomic ranks from genus to phylum based on coding gene sequence divergence. 16S rRNA gene sequences have been identified in genomes, but were not used in phylogeny reconstruction [13]. Here we demonstrate that after removal of contaminant and chimeric 16S rRNA sequences, GTDB can be used both on its own and in conjunction with collections of near full length SSU sequences and PCR amplicons to obtain improved and consistent taxonomic annotations of Archaea sequences from both metabarcoding and RNASeq.

## Materials and methods

The methods described are those used to generate version 3.1 of KSGP and GTDB+. Grant *et al.* [19] give details of methods used to generate version 1.0.

### Databases used

Results are based on GTDB version 220.0; the Sativa subset of GTDB release 207 [20, hereafter Sativa]; SILVA version 138.1 [20]; Greengenes 13.5 [10] Greengenes2 2022.10 [21]; MIDORI2 GB259, using the longest sequence for each species [22] and PR2 5.0.0 [23, 24].

### Cleaning the GTDB 16S rRNA sequence database

Initial analyses of GTDB indicated the presence of Archaea annotated as Bacteria and vice versa and contamination with Eukaryote 18S, plastid and mitochondrial sequences. The Greengenes2 backbone and SATIVA are subsets of GTDB produced by removal of misclassified sequences, but their tree-based methods require removal of short and low quality sequences [21, 25]. To allow inclusion of GTDB sequences that are short or contain gaps, we used the Ribotyper tool of Ribovore 1.0.2 [26] to identify sequences as SSU or LSU (large subunit) and assign to major taxonomic categories. Eukaryote and LSU sequences and Prokaryote SSU sequences not matching the correct domain were removed. This was followed by two iterations of RDPtools 2.0.3 *Loot* (leave one out) (https://github.com/rdpstaff/RDPTools) firstly to remove sequences incorrectly assigned at Domain level, then at Domain, Phylum or Class level followed by elimination of duplicate sequences with the *rm_dupseq* command of RDPtools.

PCR primers targeting archaeal SSU can amplify eukaryote sequences (AG, personal observation; and see results), so taxonomic databases must cover the range of eukaryote diversity likely to be present. Without this, eukaryote sequences can be incorrectly identified as highly divergent Archaea. We use eukaryote 18S and plastid sequences from the PR2 database [24] and mitochondrial sequences from MIDORI2 [22] after removing misclassified sequences using Ribotyper and a number of chimeric plastid sequences (see supplementary methods, and Table S1). We use GTDB+ as shorthand for the cleaned and deduplicated GTDB sequences concatenated with these PR2 and MIDORI2 sequences.

### Annotating the Karst *et al.* and SILVA archaea and bacteria sequences

Karst *et al.* [17, ENA accession GCA_900214305] provide an unannotated collection of >1 million sequences longer than 1200 bp consisting primarily of bacterial, archaeal and eukaryote SSU rRNA (hereafter KARST). 101 537 were identified as LSU by Ribotyper and removed. Sequences annotated as bacteria and archaea were extracted from the Ref_NR99 SILVA SSU database [20]. We refer to the combined database of GTDB+ and the (re)annotated Karst and SILVA sequences as KSGP (***K***arst, ***S***ilva, ***G***TDB, and **P**R2).

Taxonomic assignments of these were made using GTDB+ combined with three different approaches. The most conservative (*KSGP LCA*) used lowest common ancestor (LCA) assignments with USEARCH local matches to GTDB+ (minimum identity 75%; gap extension penalties 10 internal, 0 external; minimum query coverage of 25%, maxaccepts 100 maxrejects 200) and LCA v0.24 of LotuS2 with default parameters. The next most conservative *(KSGP Sintax*) carries out taxonomic assignments using SINTAX [27] with a probability cut-off of 80%. SINTAX is a high accuracy classifier [28] which is more tolerant of database sequences of varying lengths than approaches based on phylogenetic tree construction.

Finally *(KSGP+*), all sequences in KSGP were clustered at 98.5% similarity using the UClust algorithm of USEARCH after sorting by sequence length. Each cluster groups together sequences falling within a hypersphere with a radius of 1.5% divergence from its centroid, approximating a diameter of 3% divergence and 97% average nucleotide identity (ANI). This definition of a “species” is incorporated into LotuS2, but is not intended as indicating the “correct” similarity threshold to define prokaryote “species” [c.f. [29]]. These “species” were then clustered hierarchically at “genus”, “family”, “order”, “class”, “phylum”, and “kingdom” level using cluster radii of 2.5%; 3.5%; 4.5%; 6%; 11%, and 12.5%, approximating 95%; 93%; 91%; 88%; 78%, and 75% ANI, respectively. Each group at each taxonomic level is characterized by the longest sequence in that cluster. Karst and SILVA sequences were allocated to these putative taxa at levels where the LCA algorithm was unable to resolve taxonomy. Every database sequence then has a full set of taxonomic assignments from phylum to species level. These will be GTDB taxa where LCA yields a robust result and the sequence identifier of a cluster centroid where it does not. Database sequences which match an OTU at the same sequence similarity may have annotations which move from GTDB taxonomic categories to putative taxon clusters at different taxonomic levels, causing most LCA algorithms to stop classifying at this point even if the retrieved database matches are assigned to the same taxa at levels below this point. So KSGP+ *must* only be used with annotation algorithms based on the single best database match. An OTU is not assigned to a putative or GTDB taxon at a particular level if the nearest database match is below the same sequence identity threshold for that taxonomic level used in the LCA analysis in LotuS2 and indicated by the symbol “?” in output. This enables the user to readily see which elements of a taxonomic assignment are based on matches to GTDB; which are based on similarity to database sequences from taxa that are either undescribed or are not yet represented in GTDB; and which represent novel phylogenetic diversity not yet present in the databases being used. Subsequent references to SILVA are to version 138.1 of Ref_NR99 SILVA SSU with its original annotations.

### Example metabarcoding and metatranscriptomic data sets

Archaea metabarcoding and RNASeq data were generated using DNA and RNA extracted from estuarine sediments along a metal pollution gradient [30].

### Sample collection, DNA & RNA extraction

DNA and RNA were extracted from 1.4–2.7 g of 45 intertidal sediments [locations in 30] using the RNeasy PowerSoil Total RNA kit and DNA elution accessory kit (Qiagen, Hilden, Germany) following an optimized version of the manufacturer’s instructions with an added heat block step (45°C for 15 min) prior to the solution being added to the column. Nucleic acids were quantified using Invitrogen Qubit RNA and dsDNA broad range kits (ThermoFisher, Loughborough, UK) measuring fluorescence with either a qPCR machine or a Qubit 4 fluorimeter.

### PCR and sequencing

PCR amplification was carried out using the SSU1ArF/SSU520R primer pair [7] with Illumina sequencing adapters, barcodes and length heterogeneity spacers appended to their 5′ ends [following [31]]. Cycling parameters were an initial denaturation at 98°C for 10 min, followed by 35 cycles of denaturation at 98°C for 30s; annealing at 50°C 30s; extension at 72°C for 30s and a final extension at 72°C for 5 min. Sequencing was carried out using a single Illumina Novaseq SP flow cell with 250 bp paired end reads, in combination with PCR generated libraries for amplicons of similar length for several other target genes from the same sites.

### Total RNA library prep and sequencing

Sequencing libraries were prepared from total RNA using a Corall total RNA-seq library preparation kit (Lexogen, Vienna, Austria) following the manufacturer’s instructions. Sequencing was carried out using a single Illumina MiSeq nano flow cell, with 250 bp paired end reads.

### Bioinformatics

The bulk of analyses were carried out using the LotuS2 pipeline [32]. Analysis of sequence data, including OTU and tree construction and taxonomic annotation with an individual database, required a single command and was completed in under 2 h on a 64 core Intel Xeon computer. Sequences were demultiplexed and cleaned [33, 34]; OTUs were clustered at 97% similarity using UPARSE [35]; zero radius OTUs (zOTUs) were identified using UNoise [36] and reads mapped to OTUs/zOTUs using Minimap2 [37]. Database coverage was assessed by finding the best single match to each OTU using the UBLAST option of USEARCH ([38], with an E-value of 0.05) in SILVA, Greengenes, Greengenes2, GTDB, SATIVA, KARST and KSGP. Matches in the NCBI nt database as at 29 April 2024 were found using the BlastN executable version 2.14.0 [39] with an E-value cut-off of 0.05 and a query coverage cut-off of 50%. Database coverage was visualized by plotting sequence similarities of the best match in descending order; coverage of pairs of databases was compared using scatter plots of these similarities with density contours superimposed.

Taxonomic assignments from species to phylum were carried out using USEARCH_local searches against the SILVA, GG2, GTDB+, and KSGP databases followed by LCA v0.24 within LotuS2, using default parameters. Best matches for individual RNAseq reads in KSGP were found using USEARCH_local with an identity cut-off of ≥0.75 and minimum query coverage of 0.3 (equivalent to a 75 bp match).

Data handling, graph plotting and statistical analyses were carried out using R version 4.4.1 and the Phyloseq, ggplot2 and ggtree libraries [40–43]. Subsetting and interconversion of fasta and fastq files was carried out using SeqKit [44]. Sequencing reads are deposited at ENA (project accession PRJEB65254). Scripts used to generate the database and graphs are available at https://github.com/AGrantUEA/KSGP

## Results

### Congruence of 16S rRNA sequences with GTDB taxonomy based on coding genes

Ribotyper identifies 210 of 10 186 “Archaea” sequences in GTDB as bacterial in origin, often representing contamination of MAGs with 16S rRNA sequences from co-occurring organisms. In addition, some “Bacteria” sequences in GTDB are assigned to Archaea or eukaryotes, including plastids and mitochondria. After removing LSU and bacterial and archaeal SSU sequences misclassified at domain, phylum or class level and deduplicating the remainder, 178 348 unique sequences remain (further details in supplementary materials).

### Sequencing success and OTU construction

20 million sequences were used for OTU construction. UNoise generated 25 188 zero radius OTUs (zOTUs), compared with 15 179 OTUs generated by UPARSE. Around 52% of zOTUs were 100% identical to individual UPARSE OTUs, whilst 94% had at least 97% sequence similarity. The most abundant three OTUs represented 11%, 6%, and 5% of assigned reads, whilst the comparable figures for zOTUs were between 0.83% and 0.7%. Below we use OTUs, but recognize ASVs/zOTUs may be preferred for some purposes (see Box 1).

Box 1
A recommended strategy to annotate Archaea OTUs.

**Figure 1:**
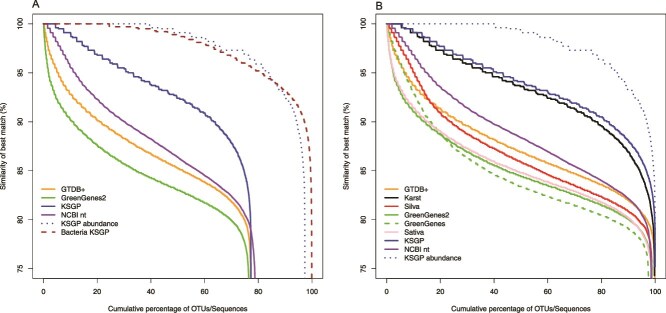
Database coverages. (A) Percentage similarity of all individual OTUs to best hit in Greengenes2, GTDB+, NCBI nt, and KSGP sorted in descending order and plotted against cumulative OTU rank (solid lines) or cumulative OTU abundance (dotted lines). Similarities for bacterial OTUs from the same environment are plotted for comparison (brown dashed line). (B) Comparable plot of sequence similarities after excluding unclassified OTUs. Details as (A) plus Greengenes, sativa, SILVA, and Karst databases.

**Figure 2:**
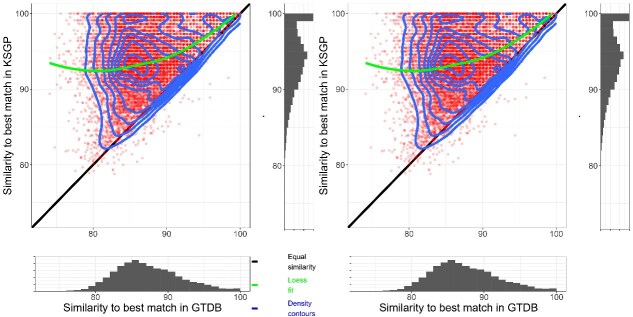
(A) Similarity of the best match in KSGP (y-axis) against similarity of the best match in GTDB+ (x-axis). The scatter diagram has density contours superimposed (blue), with marginal histograms depicting density of matches against single databases. Straight line (black) indicates identical similarity in both databases; curved green line is loess fit to the data. (B) Best matches in SILVA (y-axis) plotted against those in GTDB+. Other details as (A).

Based on the analyses presented above, we recommend the following approach to the analysis and taxonomic annotation of archaeal metabarcoding data:

1. Use UPARSE (or another similar approach) to generate 0.97 similarity radius OTUs, unless there are a priori reasons for focussing on strain-level patterns and using ASVs/zOTUs.
2. The taxonomic database used *must* cover eukaryotes and bacteria to distinguish OTUs from these domains from divergent archaeal sequences.
3. Examine the extent to which GTDB+, KSGP (and SILVA if desired) cover the phylogenetic diversity represented using the approaches in Figs 1 and 2.
4. Use KSGP LCA (or GTDB+) to carry out conservative assignments to the GTDB taxonomy.
5. Use KSGP to provide more comprehensive taxonomic assignments based on environmental SSU sequences annotated with the GTDB taxonomy, recognizing that assignments that are substantially better than those using GTDB alone should be prefaced by c.f. and that phyla, classes and orders that are not resolved in this process may represent undescribed taxa, which can be investigated further using the options below.
6. For many users, the phylum level assignments from either (4) or (5) and removal of un-classified and non-archaeal sequences will be all that is required.

Additional options.

Examine OTUs without database matches, carry out NCBI nt searches on the commonest and compare ML trees with and without these OTUs. If substantial numbers of common (low numbered) OTUs do not have database matches, these should be examined in more detail to understand what they represent.
Use KSGP+ to assign OTUs to GTDB taxa where possible and to unnamed putative taxa at levels where it is not. The cluster centroids provide a relatively long sequence for more detailed characterization of these putative taxa, including Blast searches against NCBI nt; placement of the taxon on an SSU based phylogenetic tree or design of probes to facilitate isolation and sequencing of individual cells using approaches such as fluorescence-activated cell sorting [60].
Total RNAseq using short reads provides estimates of the relative abundance of taxa without PCR bias so is a very valuable addition to metabarcoding. Long rRNA sequences obtained using a linked read approach similar to that used by Karst *et al.* or PacBio reads of cDNA prepared from total RNA would facilitate the approach that we have used here for environments where equivalent data to Karst *et al*. are not available.

### Database coverage

We can view OTUs as scattered throughout a hypervolume. For a particular search radius, the number of OTUs with a database match indicates the proportion of the hypervolume covered by the database. This proportion varies between 75.3% and 77.3% for the Greengenes, Greengenes2, GTDB+, SILVA, KARST and KSGP databases, with a slightly higher proportion for NCBI nt (Table 1). OTUs without a match could represent uncharacterized phylogenetic diversity or PCR/sequencing artefacts. The number of unique reference sequences that are the closest match to an OTU indicates how densely database sequences are distributed within this hypervolume, varying from 618 for Greengenes2 to 4536 in KARST and 4624 in KSGP (Table 1). Matches in NCBI nt fall between these extremes, with 2947 unique accessions. Cumulative plots of similarities to the closest match (Fig. 1) provide more detail on proximity of matches. SILVA has more matches at high similarity (>90%) than GTDB+, but GTDB+ performs slightly better at lower similarities indicating that it captures a greater proportion of overall phylogenetic diversity. If phylogenetic coverage were uniform, the number of unique reference sequences should be greater for larger databases. However, this is lowest for the largest database, Greengenes2, presumably resulting from stringent pruning of GTDB during the construction of the Greengenes2 backbone. There are many more high similarity matches in the >1 million sequences provided by Karst *et al.* [17, black line in Fig. 1] than in NCBI nt (purple), with the full KSGP database (blue) performing only slightly better than this. Karst *et al*. [17] report that their data contains 61 266 archaeal sequences, which cluster into 3410 OTUs at 97% similarity, but note that many of these have relatively low similarity to previously detected SSU sequences. The bulk of these novel sequences are from “sediments” (seven estuarine and marine and five freshwater). Inclusion in KSGP of the KARST sequences means that KSGP provides much better coverage than all other databases with the exception of a small number of OTUs which have a match only in NCBI nt. The SATIVA, Greengenes and Greengenes2 databases perform more poorly than either GTDB+ or SILVA.

**Table 1:** Coverage of Archaea OTUs by the different databases as indicated by the percentage of OTUs for which a Blast match was found and the number of unique accessions in the database represented amongst these hits.

Database	Percentage of OTUs with a Blast hit	Number of unique accessions with highest similarity to an OTU
KSGP	77.3	4962
KARST	77.1	4536
NCBI nt	78.8	2587
SILVA	77.1	1571
Greengenes	75.3	1134
GTDB+	77.2	1017
SATIVA subset of GTDB	76.3	666
Greengenes2	76.5	618

When plotted by cumulative sequence abundance rather than OTU rank order (blue dotted lines in Fig. 1), over 95% of individual Archaea sequences have a match in KSGP at >90% similarity, showing that common OTUs are more likely to be successfully annotated than rare ones. By contrast, bacterial OTUs from the same sediments almost all have matches in GTDB+ at higher sequence similarity than do Archaea (Fig. 1, red dashed line), illustrating our much greater understanding of bacterial phylogenetic diversity.

Figure 2 provides more detailed comparison of taxonomic coverage between the GTDB+ and both SILVA and KSGP. The modal similarity with GTDB+ is 85% (Fig. 2A) and that for SILVA a little lower, although SILVA also contains more high similarity matches than GTDB+ (Fig. 2B). Similarities with KSGP have modes at 95% and ≥ 99%, showing that the KARST sequences match a substantial fraction of the phylogenetic diversity in our samples at family/genus and species level respectively and suggesting that the SSU1ArF/SSU520R primer pair successfully amplifies a wider range of phylogenetic diversity than do older PCR primers used to amplify many environmental Archaea sequences present in SILVA.

### OTUs without database matches

**Figure 3:**
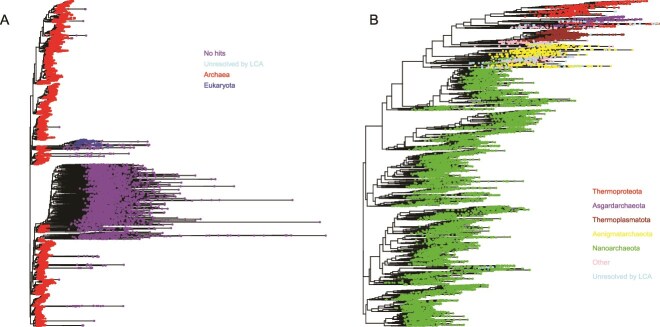
(A) Maximum likelihood tree of all OTUs classified at domain level. “Unresolved by LCA” do have matches in KSGP, whilst “No hits” do not and are likely to be PCR artefacts (see text). (B) Maximum likelihood tree after removing OTUs without a database match (“No hits” Fig. 1A) or identified as eukaryotes. The five dominant phyla are indicated. Other phyla are grouped together (blue) and archaea where taxonomy is not resolved at phylum level by LCA are indicated in pink.

Of 15 179 OTUs, 22.8% (3456) did not have matches in KSGP. Of the remainder, 11 450 (75.4%) are identified as Archaea using KSGP Sintax, 1.7% are eukaryotes, including the 51st most abundant OTU and 13 of the 1000 commonest and 0.1% did not have their domain resolved. OTUs without database matches could represent novel phylogenetic diversity; organisms other than Archaea and/or sequencing/PCR artefacts. The great majority are rare and none are within the most common 1000 OTUs. The bulk are located on long branches of the maximum likelihood tree (Fig. 3A), forming a large group just below the centre and had only short (20–30 bp) matches in NCBI nt, suggesting they represent sequencing errors or PCR artefacts. Removing these OTUs shifts the cumulative similarity curves rightwards, but has little impact on their relative positions (Fig. 1B).

### Archaea taxonomic annotation performance of KSGP

**Figure 4:**
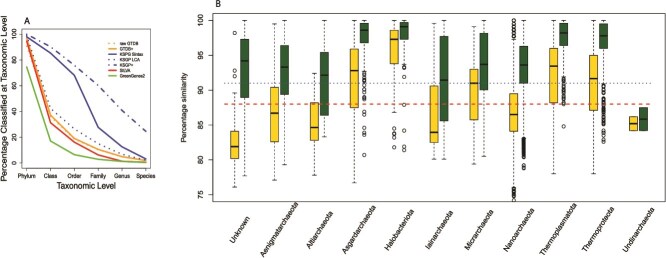
Taxonomic assignments. (A) Percentages of archaeal OTUs with taxonomic assignment at levels from phylum to family based on four different databases—KSGP, GTDB+, and SILVA. In addition to the standard version of KSGP, results are presented for KSGP+ (dot/dash blue line) and the LCA annotated version of the KSGP database (dotted blue line). (B) Box plot of sequence similarities to closest match to individual OTUs in GTDB+ (yellow) and KSGP (green), broken down by archaea phylum as assigned using KSGP. Blue dotted and red dashed lines indicate 91% and 88% sequence similarity, the cut-offs used within LotuS2 for order and class level assignments respectively. Phyla with fewer than 10 OTUs are excluded.

Excluding OTUs without a database match eliminated almost all long branches (Fig. 3B). The number of OTUs identified as Archaea was similar for KSGP Sintax, KSGP+, KSGP LCA, GTDB+, and SILVA. Using KSGP Sintax, 98.0% of KSGP Archaea were assigned to phylum, 85.3% to class and 66.1% to order (Fig. 4A). Using SILVA, only 94.6% were assigned to phylum with substantially lower proportions for taxonomic levels below this (by a factor of 2.7 for Class and 4.2 for Order). This reflects the presence in KSGP of many more high similarity matches than GTDB, particularly for Altiarcheota, Iainarcheota, Nanoarcheota, and OTUs not assigned to a phylum (Fig. 4B). When GTDB was used without database cleansing, only 91% of OTUs could be classified at Phylum level and this figure reduced to only 75% for the GreenGenes2 database, with taxonomy not resolved at Domain level for 12% and 6% misclassified as bacteria.

KSGP LCA assigns 99% of OTUs to phyla, but performs only a little better than GTDB+ at lower taxonomic levels, so some of the improvement obtained using KSGP may reflect assignment of OTUs from unnamed lineages to the nearest named taxon at the same level. KSGP+ achieves higher classification success at all taxonomic levels, including 60 and 41% at family and genus level (Fig. 4A). These clusters are likely to represent phylogenetically distinct uncharacterized lineages, as noted by Karst *et al.* [17]*.* For example, 19 OTUs are assigned to a phylum level cluster and 95 to a class level cluster, with OBEP011221528 and OBEP011253457 from the Karst *et al.* data as their respective centroids.

For OTUs not assigned to phylum using KSGP, the median sequence similarity of the nearest match increases from well below the 88% threshold for classification at Class level using GTDB+ to around the 93% threshold for assignment to families using KSGP. The level of annotation success for an individual OTU depends upon both the sequence similarity of the nearest matches and the level to which these matches are annotated. Although SILVA has a greater number of high-similarity matches than GTDB+, many of these have limited taxonomic information. By contrast, all sequences in GTDB are annotated to species level and as a result, LCA analysis based on GTDB+ has a slightly higher success rate than that based on SILVA (Fig. 4A).

We examine the taxonomic assignments of our two commonest OTUs to illustrate what lies behind these improvements. Within GTDB+, our most abundant OTU has 100% sequence similarity with four *Nitrosopumilus* species, and is placed in this genus using GTDB, KSGP and KSGP+. The second OTU has 92% sequence similarity to a species of Thermoproteota (*Bathyarchaeum*) in GTDB+ and is assigned to order Bathyarchaeales on this basis. It assigned to family Bathyarchaeacea using KSGP Sintax on the basis of 97% similarity to 7 sequences in this family. KSGP+ assigns it to putative genus and species centred on the longest of these, a near full length 16S rRNA sequence (EU420690, from a coastal wetland) to which it has 97.3% similarity.

KARST also includes information on the environment from which each sequence was retrieved. The great majority of our OTUs have a closest match to a sequence from sediments and 45% of these are from one sample sd04, a muddy sediment from Limfjorden, Denmark. The great majority of their archaeal sequences are from sediments and the largest number of these are from sample sd04. However, when standardized for database coverage our OTU sequences show significantly more matches than expected with many of Karst *et al.’s* (2018) marine sediment samples, including sd04, and significantly fewer matches with freshwater sediments and an anaerobic digestor. Surprisingly, an estuarine sediment (sd10) also has significantly fewer matches.

**Figure 5:**
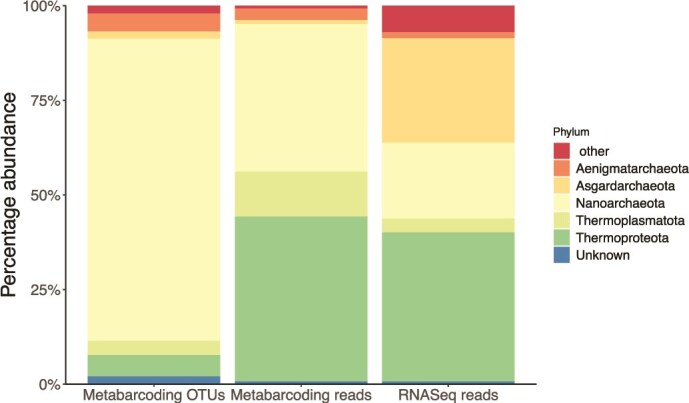
Phylum assignments of archaeal sequences from estuarine sediment samples using KSGP, after excluding eukaryotes, bacteria and OTUs without a database match. Columns are metabarcoding data annotated by proportion of OTUs and proportion of reads, both of which are subject to PCR primer bias, and proportion of RNAseq reads.

In our metabarcoding data, 79.8% of archaeal OTUs are assigned to the phylum Nanoarchaeota (Fig. 5), 70.7% to class Nanoarchaeia and 47.9% to order Woesearcheales. However, the relative abundance of these OTUs is lower than for those in other phyla, so Thermoproteota and Thermoplasmatota make up a greater proportion of sequences (Fig. 5).

### RNASeq results

We obtained 537 330 RNA reads. Ribosomal depletion was not used, so most represent rRNA, predominantly SSU and LSU in approximately equal numbers [45]. Archaea make up a rather small proportion of annotated reads (436) compared to bacterial (189309) and eukaryotic (80678) SSU sequences. In relative terms, ⁓0.23% of prokaryote SSU sequences and 0.16% of annotated sequences were of archaeal origin, lower than the 0.5% of all SSU sequences previously reported for soil [45]; the 0.8% of all PCR amplicons for boreal lakes [46]; the 4% of prokaryote RNA sequences in Karst *et al.* [calculated from data in 17] or the 8% of rRNA genes in oceanic plankton samples [47]. Thermoplasmatota make up a similar proportion of reads in the RNASeq data and metabarcoding sequences, but Nanoarchaeota are less abundant whilst Asgardarchaeota are more common (Fig. 5).

## Discussion

Curated reference databases play a crucial role in metabarcoding and metatranscriptomics but there are significant shortcomings with current taxonomic databases for Archaea. After removing PCR artefacts, only 37% of our Archaea OTUs had a match in SILVA at >88% sequence similarity expected within a Class. In addition, many of the sequences in SILVA and other databases are relatively poorly annotated because phylogenetic tree construction can be a significant challenge if only a partial 16S rRNA sequence is available. Similarities with GTDB+ are a little higher and all GTDB sequences have a full taxonomic annotation based on coding genes so both taxonomies and phylogenies will be more robust than those based on 16S rRNA genes only, although the 16S rRNA genes in GTDB require database cleansing before use (see the next paragraph). Further, GTDB is being regularly updated, whereas the most recent full release of SILVA dates from 2019 (https://www.arb-silva.de/) and at the time of writing the RDP website is no longer operational (http://rdp.cme.msu.edu/). Greengenes was last updated in 2019, although has been superseded by Greengenes2 (https://greengenes2.ucsd.edu/) in 2023. For all these reasons, GTDB offers a more future proof basis for taxonomies and phylogenies, but fully realizing this potential will require genome sequence data from representatives of clades that are currently poorly characterized.

Processing of genomes by GTDB includes checks for contamination in the coding genes used for phylogeny reconstruction [48] but not in the associated SSU sequences. As a result, a number of GTDB SSU sequences are not congruent with their GTDB classification, some at domain level. Without polishing to remove these, 5% of archaeal OTUs could not be assigned to a domain and a further 4% were not assigned to phyla. GreenGenes2 and The SATIVA subset of GTDB [20] remove misclassified sequences but their use of tree-based methods requires exclusion of sequences that are short or contain ambiguities and removes many archaeal MAGs. In consequence SATIVA and GreenGenes2 had a substantially poorer coverage of our OTUs, reducing the success of taxonomic assignments, with GreenGenes2 performing particularly poorly. So Greengenes2, Sativa and an unmodified GTDB cannot be recommended as taxonomic references for metabarcoding or metatranscriptomics of Archaea. Archaeal primers often amplify some eukaryotic SSUs so prokaryotic databases should be supplemented with a eukaryote database [c.f. [49, 50]].

Significant phylogenetic diversity of Archaea remains to be fully characterized, certainly at the level of class and perhaps phylum [18, 51]. Karst *et al*. identify eight deeply branching lineages of Archaea in their RNASeq data [17]. Our analyses display the extent to which GTDB and even SILVA fail to fully capture the phylogenetic diversity present in our samples (Figs 1 and 2). This challenge can be partially overcome by using the consistent GTDB taxonomy to annotate large sets of environmental rRNA gene sets such as that published previously by Karst *et al.* and reannotate prokaryote sequences from SILVA. Annotating using a k-mer matching algorithm such as SINTAX [27] rather than the phylogenetic tree based approaches of SATIVA [25] or Greengenes2 [21] allows us to incorporate information from incomplete SSU sequences, leading to a substantially higher level of classification success. KSGP and GTDB+ allow us to identify the great majority of archaeal OTUs to phylum level whilst also providing a strategy to exclude PCR artefacts and bacterial OTUs. KSGP also allowed us to substantially increase the number of annotated OTUs to class and order level. After PCR artefacts are excluded, KARST contains sequences with >90% sequence similarity to ˃80% of our OTUs suggesting that the great majority of our OTUs are in the same class, order or even family as particular sequences in KARST [52]. We are not able to annotate all of Karst *et al.’s* deeply branching Archaea, but this will improve as genomes from deeply branching lineages are added to GTDB over time. The use of an LCA strategy within LotuS2 [32, 52] will likely remove false positive assignments to KSGP, but taxonomic annotations of database sequences within poorly characterized parts of the phylogeny may be less robust than those achieved with KSGP LCA or directly with GTDB+. Assignment of an unknown sequence to a particular taxon using LCA requires that most database sequences from that taxon match the query at the appropriate level of similarity for that taxonomic level. By contrast SINTAX classifies sequences based on relative numbers of k-mers shared with different taxa. If the query sequence is equidistant between two clades, the two approaches will give similar results. But if the database contains only one nearby clade, LCA will classify the unknown sequence as far the taxonomic level at which sequence similarity drops below the threshold being used for that level. However SINTAX is likely to classify the unknown sequence to a lower taxonomic level than this as it will share more k-mer matches with the nearby clade than other more distant clades. So we can be less confident in the names that KSGP assigns to these taxa. These should be interpreted as indicating a close match to a single taxon that *is* robustly placed on a phylogenetic tree rather than as a definitive taxonomic assignment. This involves an element of “lumping” [53] and it may be wise to preface them with “c.f.” [sensu [54]] to indicate this. For abundant OTUs one can examine in more detail levels of sequence identity with the closest matches in both GTDB+ and KSGP, as illustrated above for our two commonest OTUs. Such classifications are, nevertheless, very useful to guide more detailed study of the components of the community. In our case, the relatively high diversity found in Nanoarchaeota, and order Woesearcheales in particular is intriguing, and the much higher sequence similarities for this phylum in KSGP than in GTDB+ (Fig. 4) suggests that phylogenetic diversity at the level of class and order within this phylum is not yet captured by GTDB and the MAG-centric studies it relies on. The same strategy that we have used with the Karst data could be used to add other near full-length sequences to a custom database or to provide an independent check on taxonomic assignments in existing databases. We have chosen to use a threshold probability of 0.8 when annotating the SILVA and Karst *et al.* sequences, but a more stringent probability may be preferred by some. Failure to assign an OTUs to a known taxon at a particular level can result from absence of a sufficiently close sequence match in the database or from incomplete taxonomic characterization of sequences that *are* present. KSGP+ annotations make it straightforward to distinguish the two and in the latter case, provide the longest possible sequence for further taxonomic characterization (see Box 1, additional options b).

The SSU1ArF/SSU520R primer pair was designed to amplify as broad a range of Archaea, but there is likely yet phylogenetic diversity that is still being uncovered [55–57] and the RNASeq data suggest this primer pair may generate PCR biases against Asgardarcheota and in favour of Nanoarcheota, (or that the former are metabolically more active than other Archaea). We recommend that RNAseq is carried out alongside metabarcoding to give a less biassed taxonomic representation of a community and the relative importance of Archaea, Bacteria and eukaryotes [c.f. 45]. Full length SSU RNA sequences would be the gold standard for this RNASeq work, as used in Karst *et al.* [17], but their methods are technically challenging and relatively time consuming [58]. For quantifying absolute abundance, we have shown that library preparation from total RNA using a commercial RNA kit allows us to assess the relative abundance of Bacteria and Archaea, yielding more SSU sequences than metagenomic approaches [c.f. 16, 58].

Other studies might see smaller improvements of taxonomic annotations than we achieve for our data for two reasons. Archaea make up a smaller proportion of prokaryotes in our estuarine sediments than they do in soil, freshwater, seawater and marine deep (anoxic) sediments [[59] and our RNASeq data], so may be under-represented in the MAGs which form a key component of GTDB. But more importantly, we have used PCR primers that amplify archaeal lineages that are absent or under-represented in data generated with widely used primers [7]. These lineages are poorly represented in SILVA, but are well covered by Karst *et al.*’s RNASeq data, which include many sequences from marine sediments.

The coding-gene based taxonomy established by GTDB provides a robust foundation for annotation of Archaea metabarcoding data. However, some of the 16S rRNA sequences in GTDB are not from the same organism as the coding genes used for taxonomic assignment, so cleaning of these data is required and GTDB should be combined with a eukaryote database to distinguish eukaryote 18S, plastid and mitochondrial sequences. GTDB alone gives a small improvement in the taxonomic annotation of our archaeal data. However, a much greater improvement is obtained when GTDB is used to annotate SILVA archaeal and bacterial sequences and a collection of near full length rRNA sequences to create the KSGP database. Most of this improvement comes from including the Karst *et al.* [17] sequences, indicating that future attempts to expand database coverage should focus on direct rRNA sequencing rather than on PCR amplicons with their associated primer biases, perhaps in conjunction with isolation and single cell sequencing of representatives of poorly characterized lineages [60]. This is likely to be particularly useful for environments that are not well covered in the Karst *et al.* [17] data. KSGP is also likely to facilitate annotation of bacterial metabarcoding data.

## Supplementary Material

Supplementary_Appendix_ycaf094

Supplementary_material_description_ycaf094

## Data accessibility

The GTDB and KSGP database, including the KSGP LCA and KSGP+ annotations are available at ksgp.earlham.ac.uk. Unprocessed DNA sequences for the Archaea are available as ENA accession PRJEB65254. Sequences of the Archaea and Bacteria OTUs; the raw RNA sequencing data and sequences removed from source databases during the construction of KSGP 3.1 are available at https://github.com/AGrantUEA/KSGP.
