# Supplementary material for: KSGP 3.1: improved taxonomic annotation of Archaea communities using LotuS2, the genome taxonomy database and RNAseq data
Source: ISME Commun. 2025 Jun 3;5(1):ycaf094. doi: 10.1093/ismeco/ycaf094 (PMC12203549; doi:10.1093/ismeco/ycaf094)
Supplement: Supplementary_Appendix_ycaf094 [file supplementary_appendix_ycaf094.pdf]

Supplementary material

Alastair Grant, Abdullah Aleidan, Charli S. Davies, Solomon C. Udochi, Joachim Fritscher, Mohammad Bahram and Falk Hildebrand

KSGP 3.1: improved taxonomic annotation of Archaea communities using LotuS2, the Genome Taxonomy Database and RNAseq data

ISME Communications 2025

Supplementary Methods

Although GTDB provides 16S rRNA sequences for each accession, these are not used in assignments to the taxonomic hierarchy for either Bacteria or Archaea, neither are they checked for consistency with the taxonomic assignment derived from coding gene sequences. Initial analyses indicated that some Archaeal 16S rRNA sequences were incorrectly labelled as Bacteria and vice versa and that GTDB also contains some sequences that are in fact eukaryote 18S rRNA, plastid or mitochondrial sequences. Despite receiving considerable manual curation, the PR2 and MIDORI2 databases also contain a number of mis-assigned sequences, so all need some cleansing.

Database Cleansing

Screening of databases for incorrect assignments at domain level and mis-identification of mitochondrial and plastid sequences using Ribovore

The Ribotyper program of the Ribovore suite of tools uses hidden Markov models to identify the best fitting model for individual sequences (Schäffer et al., 2021). We used this to screen sequences in GTDB, MIDORI2 and plastid and eukaryote rRNA 18S sequences from PR2, discarding sequences that did not have the as the best fit the same model as the taxonomic annotation in the original database. This removed 1% of GTDB and PR2 plastid sequences and 13% of MIDORI2 sequences. We used the same approach to remove apparent LSU sequences from the Karst et al. database

The MIDORI2 database

Phylogenomic analyses place mitochondria within the alphaproteobacterial order Rickettsiales (Wang & Wu, 2015) and some SSU databases use this taxonomic assignment. However, the sequence similarity of SSU rRNA between mitochondria and Rickettsiales genes is less than 60%. Our analyses treat 75% sequence as the minimum threshold to assign two sequences to the same phylum, so we give mitochondria the same status as a domain/"kingdom" in our taxonomic hierarchy. MIDORI2 GB259 contains 67962 mitochondrial SSU rRNA sequences (Leray, Knowlton, & Machida, 2022). Manual searches (details not presented) indicate that non-mitochondrial sequences in MIDORI2 include free-living representatives of the Rickettsiales and a number of non-mitochondrial endosymbionts such as *Wolbachia*.

The PR2 database

PR2 uses a non-standard 9-level taxonomic hierarchy consisting of Domain, Supergroup, Division, Subdivision, Class, Order, Family, Genus, and Species (https://pr2-database.org/documentation/pr2-taxonomy-9-levels/). To give the same number of taxonomic levels as in GTDB, our modified version of PR2 combines its Supergroup, Division and Subdivision into a single category equivalent to Phylum level in GTDB. The highest level we refer to as "Kingdom" rather than domain, reflecting the fact that it contains several categories alongside the domains of Bacteria, Archaea and Eukaryota. Within metazoa, clades that are almost universally given the status of Phylum are given the status Class by

PR2, so PR2 based taxonomies cannot be combined with other eukaryote taxonomies such as those used by SILVA. None of these decisions should be taken as indicating a view on the correct taxonomic status of particular labels or the equivalence of taxonomic levels between prokaryotes and eukaryotes.

Supplementary Results

The PR2 database identifies several different types of plastid sequences. In addition to 18 mitochondrial sequences these fall into four categories:

1. 22 18S rRNA sequences from apicoplasts – non photosynthetic plastids found in apicomplexans, e.g. AB471803 (Köhler et al., 1997). These sequences have high similarity and high coverage matches only to apicoplasts of closely related species and Ribotyper has apicoplast as the best matching model. They are identified as a separate "kingdom" in our version of PR2 as they are in the original.
2. Seven 16S rRNA sequences from "chromatophores" from the Amoeba *Paulinella.* These are photosynthetic plastids with an independent and more recent evolutionary origin to that of chloroplasts e.g. FJ184060 which resemble a "*Synecchococcus*-like free living cyanobacterium" (Gabr, Grossman, & Bhattacharya, 2020; Lhee et al., 2019) and have greater than 97% sequence similarity with 16S rRNA sequences from both *Synecchococcus* and *Prochloroccus*. In our "LCA aware" version of PR2, they grouped with these Cyanobacteria into order PCC-6307 and family Cyanobiaceae to reflect their recent origin within the *Synecchococcus/Proclorococcus* clade.
3. 148 18S rRNA sequences from nucleomorphs, residual nucleii found in some plastids, with two separate evolutionary origins (Moore & Archibald, 2009). Both of these lineages (one in each in cryptophytes and chlorarachniophytes) are represented in PR2 version 5.0 and are assigned to a separate "kingdom" in our database
4. 6668 sequences identified as plastids with "kingdom" annotated as Eukaryota:plas in PR2. The great majority of these are identified as chloroplast SSU sequences by Ribotyper, but a small number are from apicoplasts and there is some contamination with other non-chloroplast sequences (Table S1). Some of the remaining sequences appeared to chimeric with sections similar to bacteria other than Cyanobacteria, an issue noted by others (Robinson, Daligault, Kelliher, LeBrun, & Chain, 2022). USEARCH local (Edgar, 2010) was used to identify and remove 62 sequences with sections of more than 100bp that were more than 90% similar to non-cyanobacteria sequences in RDP training set 18. The remainder were assigned to "kingdom" Bacteria, phylum Cyanobacteria and class Chloroplast, with the taxonomy given by PR2 retained at the level of Order and below.

Table S1. Best Ribotyper model for database sequences. “Correct” taxonomic assignments for each database are in bold. Numbers in parentheses indicate GTDB sequences retained after Ribotyper screening.

	GTDB	PR2 plastids	PR2 Eukaryote 18S	MIDORI2	Karst et al
Total	863832	6 668	204 238	69 952	1 685 957
Bacteria SSU	**845 303** (842232)	33		333	1 449 829
Archaea SSU	**10255** (9835)			3	60 687
Eukarya	6 978	6	**203 364**	117	67 967
Of which Microsporidia	11		**1332**	4	9
Apicoplast	290	22		1018	3
Chloroplast	457	**6 598**	3	25	5979
Mitochondria	513	2	3	**60 891**	**15**
LSU Bacteria	8	6	3	7	101 474
LSU Eukarya			607		3
No Hit	28	1	258	7558	0

Supplementary references

Edgar, R. C. (2010). Search and clustering orders of magnitude faster than BLAST. *Bioinformatics, 26*(19), 2460-2461. doi:10.1093/bioinformatics/btq461

Gabr, A., Grossman, A. R., & Bhattacharya, D. (2020). *Paulinella*, a model for understanding plastid primary endosymbiosis. *Journal of Phycology, 56*(4), 837-843. doi:10.1111/jpy.13003

Köhler, S., Delwiche, C. F., Denny, P. W., Tilney, L. G., Webster, P., Wilson, R. J., . . . Roos, D. S. (1997). A plastid of probable green algal origin in Apicomplexan parasites. *Science, 275*(5305), 1485-1489. doi:10.1126/science.275.5305.1485

Leray, M., Knowlton, N., & Machida, R. J. (2022). MIDORI2: A collection of quality controlled, preformatted, and regularly updated reference databases for taxonomic assignment of eukaryotic mitochondrial sequences. *Environmental DNA, 4*(4), 894-907. doi:10.1002/edn3.303

Lhee, D., Ha, J.-S., Kim, S., Park, M. G., Bhattacharya, D., & Yoon, H. S. (2019). Evolutionary dynamics of the chromatophore genome in three photosynthetic Paulinella species. *Scientific Reports, 9*(1), 2560. doi:10.1038/s41598-019-38621-8

Moore, C. E., & Archibald, J. M. (2009). Nucleomorph Genomes. *Annual Review of Genetics, 43*(1), 251-264. doi:10.1146/annurev-genet-102108-134809

Robinson, A. J., Daligault, H. E., Kelliher, J. M., LeBrun, E. S., & Chain, P. S. G. (2022). Multiple Cases of Bacterial Sequence Erroneously Incorporated Into Publicly Available Chloroplast Genomes. *Frontiers in Genetics, 12*. doi:10.3389/fgene.2021.821715

Schäffer, A. A., McVeigh, R., Robbertse, B., Schoch, C. L., Johnston, A., Underwood, B. A., . . . Nawrocki, E. P. (2021). Ribovore: ribosomal RNA sequence analysis for GenBank submissions and database curation. *BMC Bioinformatics, 22*(1). doi:10.1186/s12859-021-04316-z

Wang, Z., & Wu, M. (2015). An integrated phylogenomic approach toward pinpointing the origin of mitochondria. *Scientific Reports, 5*(1), 7949. doi:10.1038/srep07949
