# Supplementary material for: KSGP 3.1: improved taxonomic annotation of Archaea communities using LotuS2, the genome taxonomy database and RNAseq data
Source: ISME Commun. 2025 Jun 3;5(1):ycaf094. doi: 10.1093/ismeco/ycaf094 (PMC12203549; doi:10.1093/ismeco/ycaf094)
Supplement: Supplementary_material_description_ycaf094 [file supplementary_material_description_ycaf094.docx]

Supplementary Material contains additional methods used to generate the GTDB+ and KSGP databases and information on the numbers of sequence removed as incorrectly annotated
